# Micropropagation and validation of genetic and biochemical fidelity among regenerants of *Nothapodytes nimmoniana* (Graham) Mabb. employing ISSR markers and HPLC

**DOI:** 10.1007/s13205-016-0490-y

**Published:** 2016-08-16

**Authors:** Lokesh Prakash, Sushil Kumar Middha, Sudipta Kumar Mohanty, Mallappa Kumara Swamy

**Affiliations:** 1 Department of Biotechnology, JNTU Kukatpally, Hyderabad, India; 2 DBT-BIF Facility, Department of Biotechnology and Biochemistry, Maharani Lakshmi Ammanni College for Women, Malleswaram, Bangalore, 560012 India; 3 Department of Biotechnology, Padmashree Institute of Management and Sciences, #149, Kommaghatta, Kengeri, Bangalore, 560060 India; 4 Department of Crop Science, Faculty of Agriculture, Universiti Putra Malaysia, Darul Ehsan, Serdang, Selangor 43400 Malaysia; 5 Department of Biochemistry, CPGS, Jain University, #18/3, 9th Cross, 3rd Block Jayanagar, Bangalore, 560011 India

**Keywords:** *Nothapodytes nimmoniana*, Cytokinin, Genetic fidelity, Micropropagation, ISSR, Camptothecin

## Abstract

An in vitro protocol has been established for clonal propagation of *Nothapodytes nimmoniana* which is an important source of Camptothecin (CPT). Elite source was identified based on the chemical potency to accumulate the optimum level of CPT. Different types and concentrations of plant growth regulators were used to study their effect on inducing multiple shoots from the explants regenerated from embryos of *N. nimmoniana*. Of these, a combination of N6-benzyladenine (0.2 mg L^−1^) and Indole-3-butyric acid (IBA) (0.1 mg L^−1^) proved optimum for differentiating multiple shoots in 90.6 % of the cultures with an average of 10.24 shoots per explant obtained within 8 weeks of inoculation. Nearly, 92 % of the excised in vitro shoots rooted on half strength Murashige and Skoog (MS) medium containing 0.05 % activated charcoal, supplemented with 1-naphthaleneacetic acid and IBA at 0.1 mg L^−1^ each. The micropropagated plants were evaluated for their genetic fidelity by employing inter simple sequence repeats (ISSR) markers. Ten individuals, randomly chosen from a population of 145 regenerants, were compared with the donor plant. The regenerated plants were also evaluated for their chemical potency using high-performance liquid chromatography (HPLC) analysis of CPT content. The true-to-type nature of the micropropagated plants was confirmed based on their monomorphic banding profiles with that of the mother plants using ISSR markers. Besides, HPLC evaluation of the CPT content confirmed the existence of chemical uniformity among the regenerated plants and the elite mother plant.

**Electronic supplementary material:**

The online version of this article (doi:10.1007/s13205-016-0490-y) contains supplementary material, which is available to authorized users.

## Introduction

*Nothapodytes nimmoniana* (Graham) Mabb. belonging to the family, Icacinaceae is a medicinally valuable tree. It is a native of Indomalaysia and Indochina. Barks of *N. nimmoniana* form an important source for cytotoxic quinoline alkaloid, Camptothecin (CPT) (Uma et al. 2008). CPT was first isolated by (Wall et al. 1966) from *Camptotheca acuminate* belonging to the family, Nyssaceae which also forms a major source for the extraction of CPT (Lorence and Nessler 2004; Sankar 2010). India’s exports in traditional healthcare products besides Indian Systems of Medicine (ISM) include a significant portion of medicinal plants and extracts. As per Export–Import (EXIM) bank (2010) report, barks of *N. nimmoniana* are included as major items of medicinal plants and extracts that are being exported from India. The current development on the manifestation and mortality rates, due to various forms of cancer worldwide, is extremely alarming (Jemal et al. 2008). Non-availability of enough anticancer drugs and the demand to satisfy current needs requires a sustainable source of CPT. CPT and its structural analogs have appeared as one of the most promising anticancer drugs. A number of CPT derivatives have already entered clinical trials against different forms of cancer. Topotecan and Irinotecan are already in the market as successful anticancer drugs (Arisawa et al. 1981; Hsiang et al. 1985; Aimi et al. 1989; Yamazaki et al. 2003). Besides exhibiting excellent antitumor activity, CPT inhibits viral functions by blocking the host cell topoisomerase I. Hence, it may be used to develop antiviral drugs against several DNA viruses. Pantaziz et al. (1999) reported the efficiency of CPT in inhibiting replication, transcription and assembly of double-stranded DNA of adenoviruses, papovaviruses, and herpes viruses, and the single-stranded DNA-containing parvoviruses.

Hence, the demand for CPT and its derivatives has reached US$ 2.2 billion in 2008 and expected to be more in the future (Sankar 2010). To meet this enormous demand, approximately a ton of raw material is required every year (Watase et al. 2004). Considering the potential global economic importance of this species, there is a need for large-scale production of quality planting materials for raising commercial plantations. In addition to the difficulty in the synthesis of CPT and its derivatives, the natural source becomes extinct due to the problems of drastic weather and excessive trade. Due to this fact, *C. acuminata* was recommended for protection by World Conservation Monitoring Centre in 2006. Likewise, *N. nimmoniana* is also under threat due to trade for medicine, loss of habitat and fire. Thus, it is red listed and recorded as endangered under IUCN status (Kumar and Ved 2000; Hombe et al. 2002). Conventional propagation studies in *C. acuminata* have not met the demand for CPT production (Sankar 2010). Various factors like fungal diseases, root rot, have limited the growth of *C. acuminate* and hence the total yield of CPT (Li et al. 2005). Also, the propagation of *C. acuminata* is limited only to sub-tropical climates and it takes a minimum of 10 years for plants to crop a stable fruit yield (Li et al. 2005; Sankar 2010). However, no reports exist on conventional propagation studies of *N. nimmoniana*. Thus, there is an emergency need to increase the source of CPT or to develop a protocol for mass propagation through tissue culture for large-scale production of CPT and their further elevation using elicitors and through genetic transformation. Though some work on in vitro regeneration of *N. nimmoniana* has been published using different explants, none of them has established protocols pertaining to the genetic and biochemical fidelity of the mature regenerants. Culture stress under in vitro conditions may cause genetic instability and somaclonal variation in the regenerants (Haisel et al. 2001). Therefore, assessment of clonal fidelity and progress in plant regeneration systems of the in vitro raised plants of *N. nimmoniana* will be of great significance. Application of molecular markers such as RAPD, ISSR to the micropropagated plants has proved beneficial for analyzing the genetic fidelity (Bhatia et al. 2011; Phulwaria et al. 2012; Singh et al. 2012; Kaushik et al. 2015). In addition, analyzing the CPT content of *N. nimmoniana* mature regenerants and the elite mother plant will further confirm the biochemical fidelity. Therefore, the present investigation was undertaken to establish an efficient protocol for rapid clonal multiplication of *N. nimmoniana* through shoots induced from embryos. Further, the genetic and biochemical fidelity among the micropropagated plants was established by ISSR and HPLC analysis.

## Materials and methods

### Plant materials

The drupes of *Nothapodytes nimmoniana* were collected from Amboli, Western Ghats, India, during March and shade dried completely. Mature tree explants were also collected from the fresh sprouts after the first rains. The plant material was subjected to quantitative analysis by employing HPLC. Based on the reports of HPLC analysis, elite seeds with the highest content of CPT were selected for further studies (data not shown).

### Surface sterilization of seeds

After de-husking, the seeds were treated with 1 % Cetrimide (Shalaks Pharma, New Delhi, India) solution for 10 min and thoroughly washed under a jet flow of tap water for 45 min. Seeds were then disinfected with 100 ppm Bavistin (BASF India Ltd. Thane, India) for 45 min, followed by 70 % ethyl alcohol for 3 min and rinsed thoroughly with sterile distilled water for several times. Finally, the seeds were surface sterilized with 0.1 % (w/v) aqueous mercuric chloride for 12 min and washed with sterile distilled water several times. Seeds were soaked overnight in sterile water and were dissected out under aseptic conditions.

### Culture medium and growth conditions

The study employed different media such as full strength MS (Murashige and Skoog 1962), ½ strength MS, full strength B_5_ (Gamborg et al. 1968), ½ strength B_5_, full strength Woody Plants Media (WPM) (Lloyd and McCown 1980), ½ strength WPM basal media and were used to initiate embryo cultures. MS basal medium supplemented with BA at different concentrations (0.05 mg L^−1^, 0.1 mg L^−1^, 0.2 mg L^−1^ and 0.3 mg L^−1^) was used to study the effect of BA on induction of adventitious shoots from embryo culture-derived explants. Also, MS medium supplemented with BA + KN (0.2 mg L^−1^ + 0.05 mg L^−1^ and 0.2 mg L^−1^ + 0.1 mg L^−1^) and BA + Indole-3-butyric acid (IBA) (0.2 mg L^−1^ + 0.05 mg L^−1^ and 0.2 mg L^−1^ + 0.1 mg L^−1^) was used to study their effect on multiple shoot induction from axillary buds. For rhizogenesis, half strength MS medium supplemented with *α*-naphthalene acetic acid (NAA) and indole-3-butyric acid (IBA) at different concentrations (0.05 mg L^−1^, 0.1 mg L^−1^ and 0.2 mg L^−1^) and in combinations (NAA + IBA) (0.05 mg L^−1^ + 0.05 mg L^−1^, 0.05 mg L^−1^ + 0.1 mg L^−1^, 0.1 mg L^−1^ + 0.05 mg L^−1^ and 0.1 mg L^−1^ + 0.1 mg L^−1^) with or without 0.05 % activated charcoal was used. All plant growth regulators were obtained from Sigma-Aldrich (USA). Salts and other chemicals were obtained from Qualigens, Glaxo and SRL, Mumbai (India). As a carbon source, 1 % (w/v) sucrose (DCM, Daurala, India) was added to the media and gelled with 0.7 % (w/v) agar–agar (Qualigens, India). pH of the media was adjusted to 5.7–5.8 using 0.1 N NaOH or 0.1 N HCl prior to the addition of agar. Plant growth regulators were added prior to autoclaving. The media were autoclaved at 15 psi, 121 °C for 20 min. The cultures were incubated at 25 ± 2 °C temperature on a 16/8 h photoperiod with 31.08 μE^−2^ m^−2^ s^−1^ illumination from cool white fluorescent tubes.

### Acclimatization of regenerated plantlets

After rhizogenesis, healthy plantlets with well-developed roots were removed from the medium and washed under running tap water to remove the adhering media. Later, they were treated with 1 % bavistin (BASF, Mumbai, India) solution to prevent any fungal infection, before being transferred to plastic pots (5 cm diameter) containing soil rite: sand: farmyard manure (1:1:1.5). Water was sprayed once in a day such that the plants are not completely drenched so as to maintain high humidity. The plants were irrigated with different root promoting factors like IBA or NAA at concentrations (0.05 mg L^−1^, 0.1 mg L^−1^, 0.25 mg L^−1^ and 0.5 mg L^−1^) after every 4 days (Data not shown). The potted plants were maintained at room temperature in a poly tunnel. After 6 weeks, the plantlets were transplanted to pots containing garden soil and kept under shade in the green house for another 2 weeks before being transferred to the field for developing into mature plants.

### Phytochemical analysis

Quantification of CPT was performed using high-pressure liquid chromatography (HPLC) (Lokesh et al. 2014). The analysis was performed on Waters chromatographic system (Model No. 2487) composed of Waters 515 HPLC pump, column reverse phase C18 symmetry (4.6 × 150 mm) with 5 μm particle size. The solvent system was obtained by mixing 600 mL of A (10 mL acetic acid in 600 mL distilled water) and 400 ml of B (200 mL acetonitrile with 200 mL methanol), then filtered and degased. The flow rate was adjusted to 1 ml/min and the detector was set at 254 nm. HPLC analysis of extracts yielded chromatograms with retention time of 7 min for CPT. Co-chromatography of extracts was also performed with standard samples of CPT.

### DNA isolation and ISSR analysis

For genetic fidelity studies, ten field transferred mature regenerated plants were chosen randomly among a population of 145 mature field transferred regenerated plants along with the elite donor mother plant of *N. nimmoniana.* Total genomic DNA of donor mother plant and the in vitro raised clones of *N. nimmoniana* were extracted from young leaf tissue (5 g) using the Cetyl trimethyl ammonium bromide (CTAB) method as described by Doyle and Doyle (1987). A set of 60 ISSR (synthesized from Sigma-Aldrich, Bangalore, India) primers were screened for their repeatable amplification with the DNA from the aforesaid plants, including the elite mother plant to assess the genetic stability of the regenerants. The number of ISSR primers selected for the analysis was done on the basis of their clear and scorable banding patterns. ISSR amplification was carried out in a total volume of 25 μL containing 25 ng/μL of genomic DNA, 6 ng/µL template DNA, 10 mM dNTPs each, 3 U/µL *T*aq polymerase (Bangalore Genei Pvt. Ltd.), 10 pmol of primers and PCR buffer containing 5 mM KCl, 1 mM Tris–HCl pH 9.0, 1.5 mM MgCl_2_, 0.1 % gelatin, 0.05 % Triton-X 100 and 0.05 % NP40. Genetic fidelity analysis using ISSR primers was performed in a thermal cycler (Eppendorf, Hamburg, Germany) programmed for initial denaturation at 94 °C for 5 min, followed by final denaturation at 94 °C for 1 min, primer annealing at 50 °C for 1 min, primer extension at 72 °C for 2 min and a final extension of 2 min at 72 °C. The same program was repeated for 40 cycles. The PCRs were repeated three times, using the same conditions to check the accuracy of the amplified products. Amplified products along with DNA ladder of 100 bp were electrophoresed in 1.2 % agarose gel containing ethidium bromide (0.5 µg/mL) using 1 × TBE buffer (Sambrook and Russel 2000). Wells were loaded with 25 µL of reaction mixture mixed with 5 µL of loading buffer. The first well (No. 1) loaded with amplified DNA sample of the mother plant and the well from 2 to 9 were loaded with amplified DNA of randomly selected tissue-cultured plants. Electrophoresis was run at a constant voltage of 60 V for 5–6 h in 1 × TBE buffer. The gels were photographed under UV light using a gel documentation system (Vilber,Lourmat, Z.I. Sud Torcy, France). Only clear and scorable bands at a particular position were considered.

### Statistical analysis

All the responses recorded were calculated on the basis of a minimum of 24 replicates in each experiment and repeated twice. The evaluation of CPT from different plant parts of *N. nimmoniana* was performed with four replicates. The experimental data were expressed as mean ± SE. The significance of difference among the various treated groups and control group was analyzed by means of one-way analysis of variance (ANOVA). The level of significance was set at *p* < 0.05.

## Results and discussion

### In vitro embryo cultures

Embryos cultured on half strength basal medium in all treatments exhibited the best response compared to full strength medium. These results were comparable to the findings of earlier studies (Srinivas et al. 2012; Stojicic et al. 2012; Myung et al. 2013). On half strength B_5_ basal medium, 92.8 % of embryos showed radical elongation and among them, 98.21 % showed complete plantlet formation as compared to control which showed only 11.66 % of radical elongation and did not form complete plantlets (Table 1). However, it is seen that most of the workers have used half strength MS basal media for initiation of embryo cultures (Alok et al. 2014; Seyed et al. 2014). Very few reports exist on the significance of half strength B_5_ media over its MS counterpart in raising embryo cultures. Half strength WPM showed least response with 48.24 % of radical elongation and 29.16 % of complete plantlet formation. The variations in response to different media can be attributed to the genotype of the plant and its culture conditions. Similarly, Jameel (2011) has reported the importance of choosing different basal salts for embryogenesis depending on the culture stage and cultivar type. WPM showed the least response, while half strength MS showed the next best response with 80 % of embryos showing radical and 82 % of which showing complete plantlet formation.

**Table 1:** Aseptic germinability of *Nothapodytes nimmoniana* embryos subjected to different treatments, placed on various media

Media	*Percentage of embryos showing radical	**Percentage of embryos showing complete plantlet formation
Control (sterile H_2_O with filter paper bridges)	11.66 ± 0.32^a^	0.0 ± 0.06^a^
MS basal	42.5 ± 0.12^c^	53.62 ± 0.52^d^
½ MS basal	80.0 ± 0.24^e^	82.0 ± 0.24^f^
B5 basal	48.43 ± 1.12^d^	57.41 ± 0.48^e^
½ B5 basal	92.8 ± 0.98^f^	98.21 ± 0.74^a^
WPM	34.91 ± 0.22^b^	30.31 ± 0.28^b^
½ WPM	48.24 ± 0.18^d^	39.17 ± 0.41^c^

The values within each column representing mean ± SE followed by same letters in superscript are not significantly different from each other (*p* < 0.0001). Data analyzed by GLM procedure with Duncan’s multiple range test (DMRT) using SAS^®^

*F* value 136.18 * at *α* = 0.05

*F* value 106.25 ** at *α* = 0.05

In comparison to B5 medium, the reduction in percentage of radical elongation and plantlet formation on MS medium may be due to higher concentration of nitrates and ammonium salts in it. In many woody plants, growth was found to be inhibited with full strength MS salts and the cell death reduces when low concentrations of nitrogen and ammonium salts are added into the media (Bonga and Von 1992). Also, the effect of nitrates on embryo induction in *Gossypium hirsutum* L. was reported with high response in the absence of ammonium nitrate in the medium (Ikram and Yusuf 2004). According to Kim and Kim (2002), the ratio of nitrates and ammonium salts with a nitrogen source plays a crucial role in cell growth and embryo induction. Likewise, the percentage of embryo induction in embryogenic callus decreases drastically with the incorporation of ammonium nitrate salt in the medium (Shanjani (2003). Hence, our findings also emphasize the use of half strength B5 medium for embryo induction instead of half strength MS medium and WPM medium.

### Induction of multiple shoot buds from explants of germinated embryos

The explants derived from the above in vitro embryo cultures were used for multiple shoot induction. After 30 days of inoculation, all the explants expressed their morphogenetic potentiality by regenerating adventitious buds. But the response varied in terms of the number of buds and percent frequency (Table 2). Axillary buds inoculated on MS medium containing 0.2 mg L^−1^ BA showed 97 % frequency of shoot bud regeneration. Hence, axillary bud was used as explant for further induction of multiple shoots. The axillary buds implanted on media without BA resulted in complete plantlet formation.

**Table 2:** Effect of BA and induction of adventitious shoots from in vitro germinated embryo explants of *Nothapodytes nimmoniana*

Explant	BA mg L^−1^	% frequency of shoot bud regeneration*
Root	0.05 0.1 0.2 0.3	42 ± 0.98^b^ 49 ± 1.2^c^ 60 ± 1.23^d^ 31 ± 0.65^a^
Hypocotyl	0.05 0.1 0.2 0.3	60 ± 1.30^b^ 72 ± 0.68^c^ 80 ± 0.94^d^ 40 ± 1.32^a^
Cotyledonary node	0.05 0.1 0.2 0.3	45 ± 1.22^a^ 59 ± 1.31^c^ 70 ± 0.69^d^ 52 ± 0.75^b^
Axillary bud	0.05 0.1 0.2 0.3	85 ± 0.69^b^ 87 ± 1.41^c^ 97 ± 1.11^d^ 34 ± 1.21^a^
Terminal bud	0.05 0.1 0.2 0.3	42 ± 0.67^a^ 69 ± 0.89^c^ 73 ± 0.99^d^ 53 ± 1.12^b^

* Mean values in a column followed by different letters are significantly different as determined at *p* = 0.05 according to Duncan’s multiple range test (DMRT) using SAS^®^

**Fig. 1:**
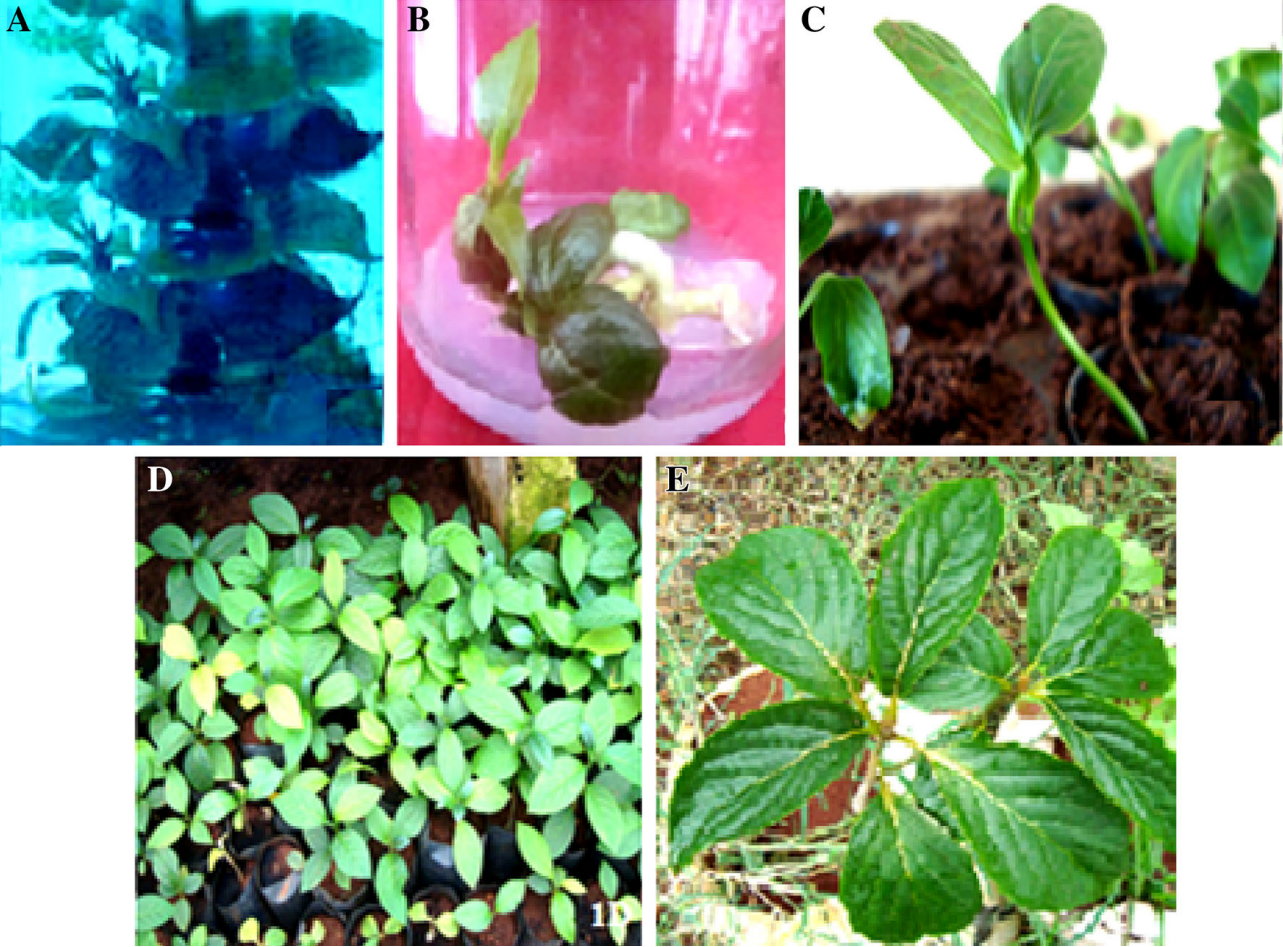
Direct organogenesis through explants of *N. nimmoniana* after 8 weeks of culture. **a** Differentiation of multiple shoots from explant after 8 weeks of culture **b** Nodal explant on MS medium supplemented with BA. **c** Rooting of in vitro raised shoot after 2 weeks of tissue culture-derived plant acclimatized to soil in glass house. **d** Acclimitization of 4-month-old tissue-cultured raised plants in garden E. Field raised elite plant of *N. nimmoniana*

Multiple shoot induction was observed from axillary buds inoculated on medium supplemented with different combinations of hormones (Table 3). Of these, BA + IBA (0.2 mg L^−1^ + 0.1 mg L^−1^) proved optimum for inducing multiple shoots in 90 % of the cultures with an average of 10.24 shoots per explant within 8 weeks of culturing. An initial bud break was also observed in the same case. (Table 3). A combination of BA with KN or IBA induced better response, though an increase in concentrations of the hormone leads to callus formation (Table 3). Direct induction from cultured seed embryos was reported in *Catharanthus roseus* (Hirata et al. 1987). However, the same was not successful in *Camptotheca acuminata* (Liu and Li 2001). In our study, we emphasize the synergistic effect of auxins and cytokinins on multiple shoot induction. Although, BA has been considered as a critical factor in multiple bud initiation, the combined effect of BA + IBA (0.2 mg L^−1^ + 0.1 mg L^−1^) was very significant as they induced more number of buds with high percent frequency (Fig. 1a). Since the discovery of synergistic effect of auxin and cytokinin by Skoog and Miller (1985), it has been used for the induction of shoot buds in plant species like *Leucaena leucocephala* (Datta and Datta 1983) and *Pterocarpus santalinus* (Lakshmi et al., 1992).

**Table 3:** Effect of plant growth regulators on multiple shoot induction from axillary buds of *Nothapodytes nimmoniana*

MS + Hormone (mg L^−1^) + AC (0.01 %)	% of cultures inducing multiple shoots*	Average no. of shoots* (mean ± SE)	Shoot length (cm)* (mean ± SE)
BA + KN 0.2 + 0.05	70.65^a^	2.45 ± 0.009^a^	1.74 ± 0.007^a^
0.2 + 0.1	74.13^b^	3.84 ± 0.008^b^	1.80 ± 0.007^ab^
BA + IBA 0.2 + 0.05	70.36^a^	6.24 ± 0.006^c^	2.24 ± 0.007^c^
0.2 + 0.1	90.61^c^	10.24 ± 0.008^d^	4.66 ± 0.106^d^

* Mean values in a column followed by different letters are significantly different as determined at *p* = 0.05 according to Duncan’s multiple range test (DMRT) using SAS^®^

Interestingly, isolated embryos implanted on MS medium supplemented with 0.2 mg L^−1^ BA and 0.1 mg L^−1^ KN showed pronounced axillary branching from the cotyledonary node region (Fig. 1b). The number of axillary branches increased with subsequent passages. Multiplication by increasing axillary branching is considered as the most appropriate way for clonal propagation of crops, ornamentals and trees with high multiplication rate and sufficient genetic stability (Lakshmi and RaghavaSwamy 1993). Similarly, enhanced axillary branching was used as a method for rapid multiplication in *Pterocarpus santalinus* and *Pterocarpus marsupium* (Anuradha and Pullaiah 1999). Therefore, induction of axillary branching in embryo cultures of *Nothapodytes nimmoniana* can offer an efficient and simple method for rapid multiplication.

### Induction of roots and hardening of plantlets

The excised in vitro shoots were transferred to half strength MS basal medium containing α-naphthalene acetic acid (NAA) or indole-3-butyric acid (IBA) at different concentrations (0.05, 0.1 and 0.2 mg L^−1^) and in combinations NAA + IBA (0.05 + 0.05 , 0.05 + 0.1, 0.1 + 0.05 and 0.1 + 0.1 mg L^−1^) with or without 0.05 % activated charcoal. Among these treatments, NAA + IBA with 0.1 mg L^−1^ each was observed to be the best treatment for root induction. A maximum of 92 % shoots induced an average of 6.23 roots with an average root length of 5.62 cm after 3 weeks on half strength MS medium (Table 4). The roots were induced directly from the shoot base without callus formation at this concentration. However, Zolman et al. (2000) report the use of IBA as the most extensively used auxin to initiate the rooting. Weisman et al. (1988) also made observations on IBA’s ability to promote root initiation, provide weak toxicity and improved stability in comparison to NAA and IAA. Further, Qaddoury and Amssa (2004) suggest that the application of IBA under in vitro conditions may induce changes in the activities of enzymes like peroxidase and IAA oxidase, which influence the accumulation of phenolic contents allowing the enactment of the favorable intracellular hormone balance for root initiation in excised shoots. In contrast, none of the shoots induced rooting on basal medium. Poor rooting response was observed with all tried concentrations of hormones in comparison to the combination of NAA and IBA (Table 4). The tissue culture-derived plantlets (Fig. 1c) were acclimatized in the field with 97 % survival. Such micropropagated plants were found to be phenotypically similar to the mother plant (Fig. 1d) and their genetic as well as biochemical fidelity was further assessed using ISSR and HPLC analysis, respectively.

**Table 4:** Effect of plant growth regulators on rooting response of *Nothapodytes nimmoniana*

MS + Hormone (mg/l)	% Root induction*	Average no of roots per shoot (mean ± SE)	Average root length (cm) (mean ± SE)
Control	**–**	**–**	**–**
NAA			
0.05	30 ± 0.1^a^	0.92 ± 0.004^a^	2.13 ± 0.012^a^
0.1	55 ± 0.1^b^	1.71 ± 0.006^b^	3.14 ± 0.013^b^
0.2	66 ± 0.2^c^	2.76 ± 0.009^c^	3.57 ± 0.016^c^
IBA			
0.05	32 ± 0.1^a^	0.78 ± 0.005^a^	1.05 ± 0.006^a^
0.1	57 ± 0.3^b^	1.98 ± 0.006^b^	1.19 ± 0.008^b^
0.2	68 ± 0.1^c^	2.11 ± 0.004^c^	3.89 ± 0.004^c^
NAA + IBA			
0.05 + 0.05	31 ± 0.1^a^	0.81 ± 0.008^a^	2.62 ± 0.006^a^
0.05 + 0.1	62 ± 0.2^b^	2.71 ± 0.004b^c^	3.21 ± 0.007^b^
0.1 + 0.05	73 ± 0.2^c^	2.64 ± 0.006^b^	3.34 ± 0.003^c^
0.1 + 0.1	92 ± 0.1^d^	6.03 ± 0.004^d^	5.62 ± 0.010^d^
NAA + AC (0.05 %)			
0.05	38 ± 0.2^a^	1.14 ± 0.005^a^	2.38 ± 0.004^a^
0.1	59 ± 0.1^b^	1.96 ± 0.009^b^	3.56 ± 0.003^b^
0.2	69 ± 0.2^c^	2.98 ± 0.010^c^	3.95 ± 0.004^c^
IBA + AC (0.05 %)			
0.05	38 ± 0.3^b^	0.89 ± 0.004^a^	1.96 ± 0.006^b^
0.1	59 ± 0.2^c^	2.34 ± 0.010^c^	1.68 ± 0.009^a^
0.2	30 ± 0.2^a^	2.61 ± 0.003^b^	4.01 ± 0.010^c^
NAA + IBA + AC (0.05 %)			
0.05 + 0.05	26 ± 0.1^ab^	0.98 ± 0.008^a^	2.98 ± 0.004^a^
0.05 + 0.1	25 ± 0.2^a^	2.96 ± 0.004^b^	3.56 ± 0.006^b^
0.1 + 0.05	25 ± 0.1^a^	3.24 ± 0.006^bc^	3.98 ± 0.007^c^
0.1 + 0.1	54 ± 0.1^c^	6.23 ± 0.009^d^	6.02 ± 0.006^d^

* Mean values in the columns are significantly different as determined at *p* = 0.05 according to Duncan’s multiple range test (DMRT) using SAS^®^

### Genetic fidelity using ISSR analysis

**Fig. 2:**
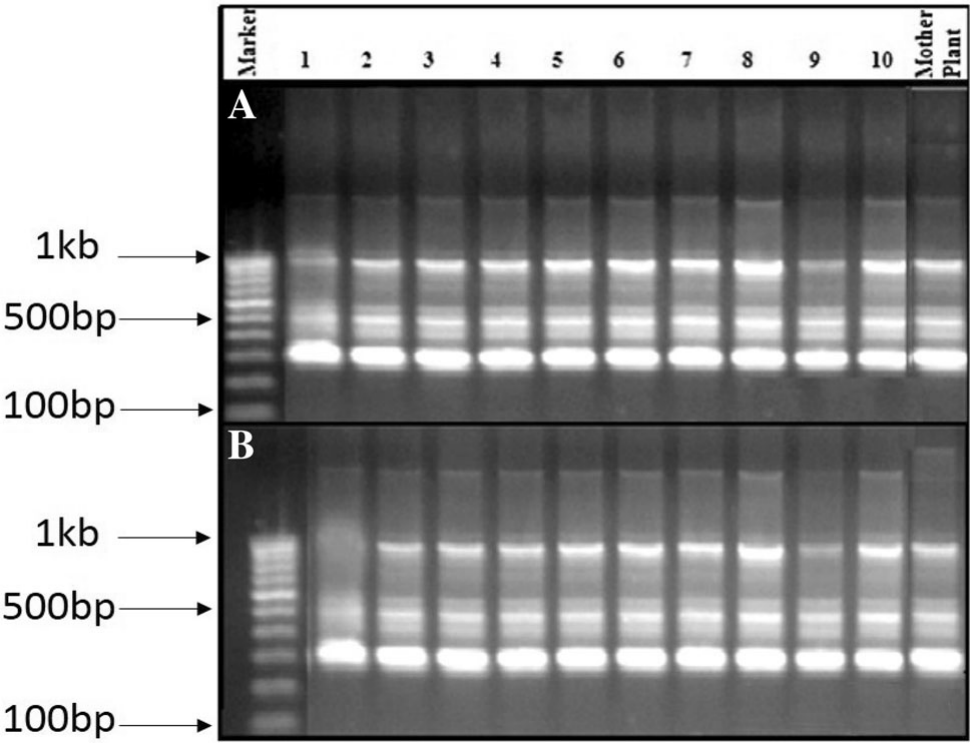
ISSR banding patterns of the in vitro raised plantlets (1–10) and mother plant of *N. nimmoniana* produced by the primers, HBIO816 (**a**) and HBIO810 (**b**) Lane Marker represents the 1 kb ladder

The genetic fidelity of in vitro propagated plants of *N. nimmoniana* was evaluated employing PCR-based fingerprinting technique. ISSR analysis was done by randomly selecting ten in vitro propagated plants and compared with the mother plant. A total of 60 ISSR primers were used for initial screening and out of which ten produced clear, distinct and reproducible scorable bands with an average of 4.2 bands per primer for a total of 33 scorable bands (Supplementary Table 1). Most of the banding profiles from in vitro propagated plants were monomorphic and similar to those of mother plant except two primers, viz. HBIO809 and HBIO810 which displayed polymorphic bands (Supplementary Table 1). The ISSR primer, HBIO816, displayed prominent monomorphic bands among the in vitro derived regenerants and the elite donor mother plant (Fig. 2a), while the primer HBIO810 displayed the polymorphic band in the regenerants (Fig. 2b). These banding profiles of in vitro propagated plants when compared with that of mother plants shown by ISSR markers indicate a greater level of genetic similarity among the regenerants and the elite donor plant of *N. nimmoniana*. According to Razaq et al. (2013) and Singh et al. (2013), the low level of genetic variation in DNA may be due to naturally occurring variation or hormonal balance, in vitro stress induced by adding biochemicals, or other nutritional conditions.

### HPLC analysis for CPT content of mature regenerants and elite mother plant of *N. nimmoniana*

**Fig. 3:**
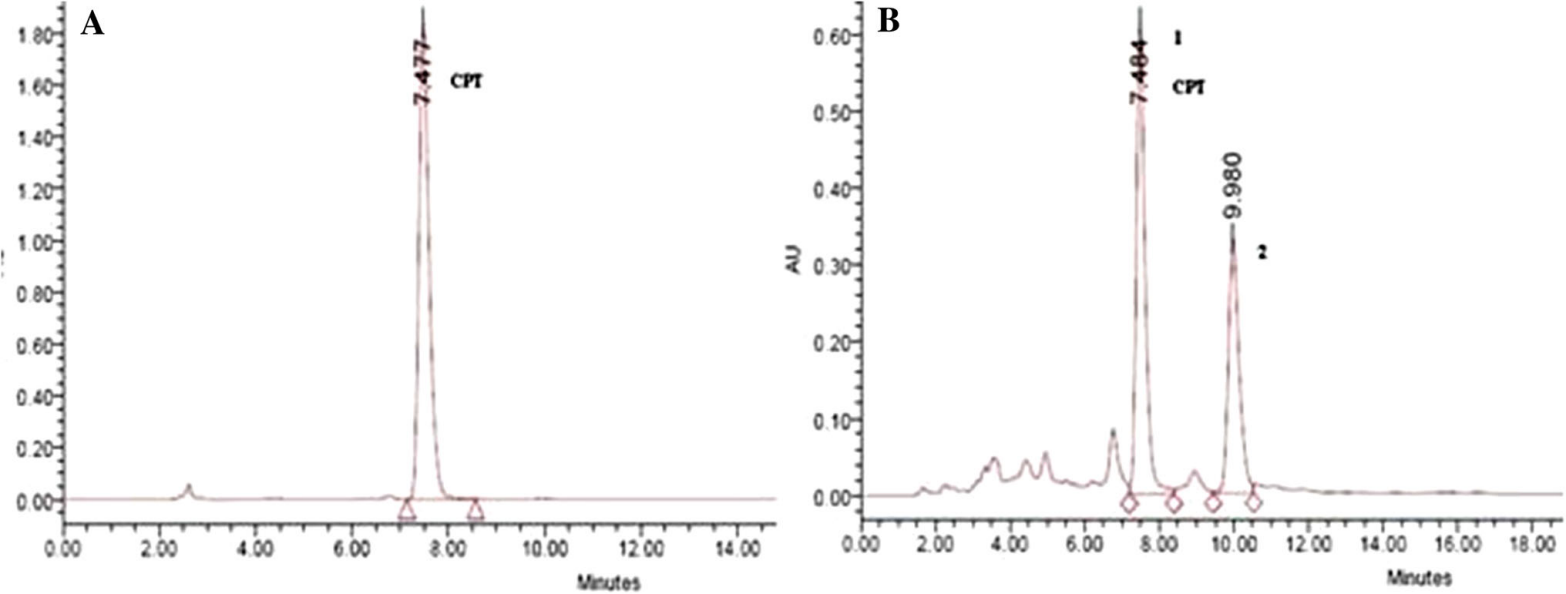
**a** HPLC chromatogram of the standard CPT. **b** HPLC chromatogram of the CPT present in the root samples of micropropagated plants of *N. nimmoniana*

The quantitative analysis of the CPT content of different plant parts, viz. leaves, stem and roots of the field raised plants of *N. nimmoniana* through HPLC revealed that the amount of CPT was variable in different parts, but was maximum in roots (0.12 % w/w) (Fig. 3). The concentration of CPT in leaves and stem was 0.0013 % w/w and 0.026 % w/w, respectively. HPLC chromatograms of CPT from the roots of elite mother stock and ten randomly selected regenerants showed consistency in retaining their chemical potency.

## Conclusion

One of the major constraints in plant-derived drugs is the low availability of active compounds which also depends upon accumulation pattern, different geographical, environmental condition and genetic makeup of the plant. This study describes an efficient protocol for rapid clonal propagation of *N. nimmoniana* using shoots derived from embryo culture. Also, the use of ISSR marker analysis revealed the genetic uniformity among the in vitro regenerants and the mother plant. Moreover, the CPT content of regenerants was found to be similar to that of the mother plant. This confirms no biochemical variations in the plants developed through tissue culture system. Therefore, this in vitro protocol can serve as a new source for obtaining large quantities of CPT compound. In addition, this clonal propagation protocol may be useful for commercial propagation to mitigate the problem of planting materials shortage as well as to conserve the elite clones of *N. nimmoniana*.

## Electronic supplementary material

Below is the link to the electronic supplementary material.

Supplementary material 1 (DOC 35 kb)

## Abbreviations

BA: N6-Benzyladenine
KN: 6-Furfuryl aminopurine
NAA: 1-Naphthaleneacetic acid
IBA: Indole-3-butyric acid
CPT: Camptothecin
ISSR: Inter simple sequence repeats
HPLC: High-performance liquid chromatography

## References

[CR1] Aimi N, Nishimura M, Miwa A, Hoshino H, Sakai S, Haginiwa J (1989). Pumiloside and deoxypumiloside, plausible intermediates of camptothecin biosynthesis. Tetrahedron Lett.

[CR2] Alok D, Sumit K, Nandeesha P, Yadav IS, Jyoti S, Chaturvedi SK, Subhojit D (2014). An efficient in vitro regeneration system of field pea (*Pisum sativum* L.) via. shoot organogenesis. J Plant Biochem Biotechnol.

[CR3] Anuradha M, Pullaiah T (1999). In vitro seed culture and induction of enhanced axillary branching in *Pterocarpus santalinus* and *Pterocarpus marsupium*: a method for rapid multiplication. Phytomorphology.

[CR4] Arisawa M, Gunasekera SP, Cordell GA, Farnsworth NR (1981). Plant anticancer agents XXI. Constituents of Merrilliodendron megacarpum. Plant Med.

[CR5] Bhatia R, Singh KP, Sharma TR, Jhang T (2011). Evaluation of the genetic fidelity of in vitro propagated gerbera (*Gerbera jamesonii* Bolus) using DNA-based markers. Plant Cell Tissue Organ Cult.

[CR6] Bonga JM, Von AP (1992). In vitro culture of Trees. Forestry Science.

[CR7] Datta SK, Datta K (1983). Auxin induced regeneration of forest trees—*Dalbergia sissoo* Roxb. through tissue culture. Curr Sci.

[CR8] Doyle JJ, Doyle JL (1987). A rapid DNA isolation procedure for small quantities of fresh leaf tissue. Phytochemical Bulletin.

[CR9] Gamborg OL, Miller RA, Ojima K (1968). Nutrient requirements of suspension culture of soybean root cells. Exp Cell Res.

[CR10] Haisel D, Hofman P, Vagneri M, Lipavska H, Ticha L, Schafer C, Capkova V (2001). Ex vitro phenotype stability is affected by in vitro culture. Biol Plant.

[CR11] Hirata K, Yamanaka A, Kurano N, Miyamoto K, Miura Y (1987). Production of indole alkaloids in multiple shoot culture of *Catharanthus rosues*. Agric Biol Chem.

[CR12] Hombe GHC, Vasudeve R, mathachen GP, Shaanker RU, Ganeshaiah KN (2002). Breeding types in *Nothapodytes nimmoniana* Graham: an important medicinal tree. Curr Sci.

[CR13] Hsiang YH, Hertzberg R, Hecht Liu LF (1985). CPT induces protein-linked DNA breaks via mammalian DNA topoisomerase I. J Biol Chem.

[CR14] Ikram UH, Yusuf Z (2004). Effect of nitrates on embryo induction efficiency in cotton (*Gossypium hirsutum* L.). Afr J Biotechnol.

[CR15] Jameel MAK (2011). Basal salt requirements differ according to culture stage and cultivar in date palm somatic embryogenesis. Am J Biochemi Biotechnol.

[CR16] Jemal A, Siegel R, Ward E, Hao Y, Xu J, Murray T, Thun MJ (2008). Cancer statistics, 2008 CA cancer. J Clin.

[CR17] Kaushik PS, Swamy MK, Balasubramanya S, Anuradha M (2015). Rapid plant regeneration, analysis of genetic fidelity and camptothecin content of micropropagated plants of *Ophiorrhiza mungos* Linn.—a potent anticancer plant. J Crop Sci Biotech.

[CR18] Kim S, Kim S (2002). Effect of nitrogen source on cell growth and anthocyanin production in callus and cell suspension culture of ‘Sheridan’ grapes. J Plant Biotechnol.

[CR19] Kumar KR, Ved DK (2000) 100 red listed medicinal plants of conservation concern in southern India. Foundation for revitilization of local health traditions (FLHT), Bangalore, India

[CR20] Lakshmi SG, RaghavaSwamy BV (1993). Regeneration of plantlets from leaf disc cultures of rosewood. Plant Sci.

[CR21] Lakshmi SG, Sreenatha KS, Sujatha S (1992). Plantlet production from shoot tip cultures of red sandal wood (*Pterocarpus santalinus* L.). Curr Sci.

[CR22] Li A, Zhang Z, Cain A, Wang B, Long M, Taylor J (2005). Antifungal activity of camptothecin trifolin and hyperoside isolated from *Camptotheca acuminata*. J Agric Food Chem.

[CR23] Liu Z, Li Z (2001). Micropropagation of *Camptotheca acuminata* Decaisne from axillary buds, shoot tips, and seed embryos in a tissue culture system. Vitro Cell Dev Biol Plant.

[CR24] Lloyd G, McCown BH (1980). Commercially feasible micropropagation of mountain laurel (*Kalmia latifolia*) by use of shoot tip culture. Int Plant Prop Soc Comb Proc.

[CR25] Lokesh P, Balasubramanya S, Anuradha M (2014). Cost effective quantification of camptothecin and a comparative study of its content in *Nothapodytes foetida* and *Ophiorrhiza mungos* sourced from selected geographical locations. Orient Pharm Exp Med.

[CR26] Lorence A, Nessler CL (2004). Camptothecin, over four decades of surprising findings. Phytochemistry.

[CR27] Murashige T, Skoog FA (1962). Revised medium for rapid growth and bioassays with tobacco tissue cultures. Physiol Plant.

[CR28] Myung JO, Myung SA, Eun YJ, Jang RL, Byung WM, Suk WK (2013). High-frequency plant regeneration from immature zygotic embryo cultures of *Houttuynia cordata* thunb via somatic embryogenesis. Plant biotechnol Rep.

[CR29] Pantaziz P, Han Z, Chatterjee D, Wyche J (1999). Water-insoluble camptothecin analogues as potential antiviral drugs. J Biomed Sci.

[CR30] Phulwaria M, Rai MK, Patel AK, Kataria V, Shekhawat NS (2012). A genetically stable rooting protocol for propagating a threatened medicinal plant—*Celastrus paniculatus*. AoB Plants.

[CR31] Qaddoury A, Amssa M (2004). Effect of exogenous indole butyric acid on root formation and peroxidase and indole-3-acetic acid oxidase activities and phenolic contents in date Palm offshoots. Bot Bull Acad Sin.

[CR32] Razaq M, Heikrujam M, Chetri SK, Agrawal V (2013). In vitro clonal propagation and genetic fidelity of the regenerants of *Spilanthes calva* DC using RAPD and ISSR marker. Physiol Mol Biol Plants.

[CR33] Sambrook JJ, Russel DDW (2000). Mol cloning lab man.

[CR34] Sankar TYD (2010) In vitro culture of *Camptotheca acuminata* (Decaisne) in temporary immersion system (TIS): growth, development and production of secondary metabolites. PhD thesis, University Hamburg, Germany

[CR35] Seyed JD, Mehrdad L, Ramin K (2014). Micropropagation of common yew using embryo culture. J Appl Biotechnol Rep.

[CR36] Shanjani PS (2003). Nitrogen effect on callus induction and plant regeneration of *Juniperus excelsa*. Inter J Agric Biol.

[CR37] Singh SK, Rai MK, Sahoo L (2012). An improved and efficient micropropagation of *Eclipta alba* through transverse thin cell layer culture and assessment of clonal fidelity using RAPD analysis. Ind Crop Prod.

[CR38] Singh SR, Dalal S, Singh R, Dhawan AK, Kalia RK (2013). Evaluation of genetic fidelity of in vitro raised plants of *Dendrocalamus asper* (Schult. & Schult. F.) Backer ex K. Heyne using DNA-based markers. Acta Physiol Plant.

[CR39] Skoog F, Miller CD (1985). Chemical regulation of growth and organ formation in plant tissue cultures. Vitro Sym Soc Exp Biol.

[CR40] Srinivas P, Samatha T, Shyamsundarachary R, Rajinikanth M, Rama SN (2012). Factors affecting germination and seedling growth of an endangered forest tree Wrightia tomentosa (Roxb.) Roem. & Schult. through in vitro Zygotic embryo culture. J Cell Tissue Res.

[CR41] Stojicic D, Janošević D, Uzelac B, Cokesa V, Budimir S (2012). Micropropagation of *Pinus peuce*. Biol Plant.

[CR42] Uma SR, Ramesha BT, Ravikanth G, Gunaga RP, Vasudeva R, Ganeshaiah KN, Ramawat KG, Merillon JM (2008). Chemical profiling of *Nothapodytes nimmoniana* for camptothecin, an important anticancer alkaloid: towards the development of a sustainable production system. Bioactive molecules and medicinal plants.

[CR43] Wall ME, Wani MC, Cook CE, Palmer KH, McPhail AT, Sim GA (1966). Plant antitumor agents. The isolation and structure of camptothecin, a novel alkaloidal leukemia and tumor inhibitor from *Camptotheca acuminata*. J Am Chem Soc.

[CR44] Watase I, Sudo H, Yamazaki M, Saito K (2004). Regeneration of transformed *Ophiorrhiza pumila* plants producing camptothecin. Plant Biotechnol.

[CR45] Weisman Z, Riov J, Epstein E (1988). Comparison of movement and metabolism of indole-3-acetic acid in mung bean cuttings. Physiol Plant.

[CR46] Yamazaki Y, Sudo H, Yamazaki M, Aimi N, Saito K (2003). Camptothecin biosynthetic genes in hairy roots of *Ophiorrhiza pumila*: cloning, characterization and differential expression in tissues and by stress compounds. Plant Cell Physiol.

[CR47] Zolman BK, Yoder A, Bartel B (2000). Genetic analysis of indole-3- butyric acid responses in *Arabidopsis thaliana* reveals four mutant classes. Genetics.

