# Supplementary material for: Micropropagation and validation of genetic and biochemical fidelity among regenerants of Nothapodytes nimmoniana (Graham) Mabb. employing ISSR markers and HPLC
Source: 3 Biotech. 2016 Aug 16;6(2):171. doi: 10.1007/s13205-016-0490-y (PMC4987634; doi:10.1007/s13205-016-0490-y)
Supplement: Supplementary file 1 — Supplementary material 1 (DOC 35 kb) [file 13205_2016_490_MOESM1_ESM.doc]

**Supplementary file:**

**Table 1: List of ISSR primers, number of scorable bands generated in *N. nimmoniana* donor plant and regenerants.**

| Sl. No. | Primer Code | Sequence (5’ 3’) | No. of scorable bands* | No. of monomorphic bands | No. of polymorphic bands |
| --- | --- | --- | --- | --- | --- |
| 1 | HBIO809 | (AG)_8_G | 3 | 2 | 1 |
| 2 | HBIO810 | (GA)_8_T | 4 | 3 | 1 |
| 3 | HBIO812 | (GA)_8_A | 2 | 2 | 0 |
| 4 | HBIO816 | (CA)_8_T | 4 | 4 | 0 |
| 5 | HBIO834 | (AG)_8_T | 3 | 3 | 0 |
| 6 | HBIO835 | (AG)_8_C | 4 | 4 | 0 |
| 7 | HBIO836 | (AG)_8_GA | 5 | 5 | 0 |
| 8 | HBIO840 | (CT)_8_TT | 3 | 3 | 0 |
| 9 | HBIO842 | (GA)_8_CG | 3 | 3 | 0 |
| 10 | HBIO864 | (ATG)_6_ | 2 | 2 | 0 |
| Total |  |  | 33 | 31 | 2 |

* only thick bands were considered
